# Cardiac biomarkers for detection of coronary artery disease in the community

**DOI:** 10.1038/s41598-024-82777-x

**Published:** 2024-12-16

**Authors:** Lars Lind, Joakim Alfredsson, Jonas S. O. Andersson, Therese Andersson, Göran Bergström, Örjan Ekblom, Erika Fagman, Tove Fall, Emil Hagström, Hannes Holm Isholth, Magnus Janzon, Tomas Jernberg, Ioannis Katsoularis, Karin Leander, Margrét Leósdóttir, Martin Magnusson, Andrei Malinovschi, Annika Rosengren, J. GustavSmith, Jonas Spaak, Per Svensson, Stefan Söderberg, Carl Johan Östgren, Gunnar Engström

**Affiliations:** 1 Department of Medical Sciences, Clinical Epidemiology, Uppsala University, Uppsala, SE 751 85 Sweden; 2 Department of Cardiology, Department of Health, Medicine and Caring Sciences, Unit of Cardiovascular Sciences, Linköping University, Linköping, Sweden; 3 Department of Public Health and Clinical Medicine, Skellefteå Research Unit, Umeå University, Umeå, Sweden; 4 Department of Public Health and Clinical Medicine, Section of Medicine, Umeå University, Umeå, Sweden; 5 Department of Molecular and Clinical Medicine, Institute of Medicine, Sahlgrenska Academy, University of Gothenburg, Gothenburg, Sweden; 6 Region Västra Götaland, Department of Clinical Physiology, Sahlgrenska University Hospital, Gothenburg, Sweden; 7 Department of Physical Activity and Health, The Swedish School of Sport and Health Sciences (GIH), Stockholm, Sweden; 8 Region Västra Götaland, Department of Radiology, Sahlgrenska University Hospital, Gothenburg, Sweden; 9 Department of Radiology, Institute of Clinical Sciences, Sahlgrenska Academy, University of Gothenburg, Gothenburg, Sweden; 10 Department of Medical Sciences, Molecular Epidemiology, Uppsala University, Uppsala, Sweden; 11 Department of Medical Sciences, Cardiology and Uppsala Clinical Research Center, Uppsala University, Uppsala, Sweden; 12 Department of Clinical Sciences Malmö, Lund University, Malmö, Sweden; 13 Department of Cardiology, Skåne University Hospital, Malmö, Sweden; 14 Departmentof Clinical Sciences, Danderyd University Hospital, Karolinska Institute, Stockholm, Sweden; 15 Unit of Cardiovascular and Nutritional Epidemiology, Institute of Environmental Medicine, Karolinska Institute, Stockholm, Sweden; 16 Hypertension in Africa Research Team (HART), North-West University, Potchefstroom, South Africa; 17 Wallenberg Center for Molecular Medicine, Lund University, Lund, Sweden; 18 Department of Medical Sciences, Clinical Physiology, Uppsala University, Uppsala, Sweden; 19 Department of Medicine Geriatrics and Emergency Medicine, Sahlgrenska University Hospital/Östra, Gothenburg, Sweden; 20 Department of Cardiology, Clinical Sciences, Lund University, Skåne University Hospital, Lund, Sweden; 21 Wallenberg Center for Molecular Medicine, Lund University Diabetes Center, Lund university, Lund, Sweden; 22 Region Västra Götaland, Department of Cardiology, Sahlgrenska University Hospital, Gothenburg, Sweden; 23 Department of Clinical Science and Education, Karolinska Institute, Södersjukhuset, Stockholm, Sweden; 24 Department of Cardiology, Södersjukhuset, Stockholm, Sweden; 25 CMIV Centre of Medical Image Science and Visualization, Linköping University, Linköping, Sweden; 26 Department of Health, Medicine and Caring Sciences, Linköping University, Linköping, Sweden

**Keywords:** Coronary atherosclerosis, Troponin, NT-proBNP, Epidemiology, Population, Biomarkers, Cardiovascular diseases

## Abstract

To investigate whether coronary artery disease (CAD) burden is associated with plasma levels of the myocardial biomarkers Troponin I (TropI) and NT-proBNP in a large population-based sample using a cross-sectional design. Coronary computerized tomography (CT) angiography was performed in 25,859 subjects without a history of atherosclerotic disease from SCAPIS study (age 50–65, 52% women). TropI and NT-proBNP were measured in plasma. Segment involvement score (SIS) was the primary exposure and TropI the primary outcome. Both SIS and coronary artery calcium score, were associated with TropI levels following adjustment for age, sex and multiple confounders (*p* < 0.001), with similar relationships in men and women. Proximal segments from all three coronary arteries were related to TropI levels independently of one another. Adding TropI to traditional risk factors marginally increased discrimination of atherosclerosis as compared to risk factors alone (C-statistics + 0.0005, *p* = 0.014). SIS was related also to NT-proBNP levels, mainly in men, but with lower estimates than TropI. The burden of CAD was related to TropI levels in both men and women. All three major coronary arteries contributed to this relationship. Adding TropI to traditional risk factors resulted in only marginally improved discrimination of coronary atherosclerosis.

**Supplementary Information:**

The online version contains supplementary material available at 10.1038/s41598-024-82777-x.

## Introduction

Troponin I (TropI) is a protein mainly found in cardiac muscle, but can also be measured in the circulation. This biomarker, along with troponin T, is widely used to diagnose patients with acute chest pain, but cardiac troponins are also reported to be elevated during other stressful conditions, such as large pulmonary embolus, tachycardia, sepsis and myocarditis.

With the introduction of high-sensitive measurement methods for TropI, detectable levels have been found in the majority of middle-aged or elderly subjects even without signs of myocardial injury or other evident cardio-pulmonary disorders^1^. Importantly, TropI levels in healthy individuals have been linked to major outcomes, such as total mortality, cardiovascular (CV) mortality and cardiovascular disease (CVD). This is seen also for troponin levels below the 99% percentile^1–4^. Hence, it has been proposed that TropI could be used for risk stratification in the primary prevention setting^5^.

Several factors have been linked to elevated TropI, such as high age, male sex, obesity, low HDL-cholesterol, elevated N-terminal pro B-type natriuretic peptide (NT-proBNP), chronic kidney disease, left ventricular hypertrophy and reduced left ventricular ejection fraction^1,2^. In a pravastatin trial, a pronounced reduction in TropI during therapy was associated with a reduced risk for future myocardial infarction^6^.

Another factor that potentially could influence TropI levels is the extent of coronary atherosclerosis. In studies including patients with coronary heart disease (CHD) without current chest pain who were evaluated by invasive coronary angiography, plasma TropI levels increased with the number of stenotic arteries^7,8^. In other studies, including patients with known CHD or chest pain evaluated by coronary computerized tomography (CT) angiography (CCTA), increasing levels of TropI were found with increasing severity of atherosclerosis^9,10^. TropI levels have also been linked to the Agatston coronary artery calcium score (CACS) in similar patient groups^11^.

Investigations of the relationship between the amount of coronary atherosclerosis and cardiac troponins in the general population free from known CHD are rare. In a Danish study that recruited 1,173 middle-aged subjects from the general population without known CHD, the presence of coronary calcium evaluated by non-contrast cardiac CT increased with increasing TropI levels^12^. Furthermore, addition of TropI to HeartScore improved the ROC-AUC by 2.4%. Although CACS is a predictor of future CVD events in the general population^13,14^, it is likely the burden of coronary artery disease, but not the amount of calcium, that drives myocardial injury. Therefore, an evaluation of TropI levels with respect to the amount and severity of coronary artery stenosis is highly warranted.

NT-proBNP is a neurohormone released by the heart in response to atrial stress with a primary function to regulate blood volume through natriuresis. This cardiac biomarker is mainly used for diagnostic purposes in suspected heart failure (HF) and in the evaluation of HF therapy. Its relationship to the amount and severity of coronary artery stenosis is likely secondary to increased filling pressure of the heart caused by myocardial ischemia. In a study of stable angina patients, NT-proBNP levels were related to the severity of coronary artery disease evaluated by cardiac CT only if a stress imaging test detected myocardial ischemia^9^.

The primary aim of the present study was to evaluate to which extent the burden of coronary atherosclerosis evaluated by CCTA was related to TropI levels in the general population free from overt CHD. For this aim, we used the Swedish CArdioPulmonary bioImage Study (SCAPIS) study with data on CCTA in approximately 25,000 individuals^15,16^. We used the segment involvement score (SIS) as the primary exposure variable, but also investigated the impact of CAC. As a secondary aim, we investigated if stenoses in some proximal coronary segments were more closely related to TropI than other segments. We also evaluated if NT-proBNP levels were related to the burden of coronary artery atherosclerosis, and if addition of TropI to traditional risk factors would increase the discrimination of coronary atherosclerosis. The main hypotheses were that the burden of coronary artery stenosis would be related to TropI levels and that stenosis in proximal segments of the coronary arteries would be of predominant importance for this relationship.

## Results

### Cardiac biomarkers vs. age and sex

Basic characteristics of the study sample are given in Table 1. TropI levels were related to higher age (Spearman rho 0.10, *p* < 0.001) and higher in men than in women (2.1 vs. 1.4 ng/L, *p* < 0.001). The 99% percentile values were 41 ng/L in men and 19 ng/L in women in the present sample. Similarly, levels of NT-proBNP were related to higher age (Spearman rho 0.19, *p* < 0.001), but, by contrast, higher in women than in men (60.0 vs. 36.9 ng/L, *p* < 0.001). TropI levels were related to levels of NT-proBNP in both men and women (Spearman rho 0.23 in men and 0.15 in women, *p* < 0.001 in both sexes).

**Table 1:** Basic characteristics of the study sample.

	Total sample (*n* = 25,859)	Women (*n* = 13,091)	Men (*n* = 12,768)
Variable	Mean (SD) or proportion	Mean (SD) or proportion	Mean (SD) or proportion
Age	57.3 (4.3)	57.4 (4.3)	57.3 (4.3)
Female sex (%)	51	–	–
Smoking status (%)	Never: 51.2	48.5	54.2
	Former: 41.2	43.5	38.8
	Current: 7.6	8.0	7.0
Education (%)	< 10 years: 9.1	8.0	10.2
	10–12 years: 45.3	42.5	48.5
	> 12 years: 45.6	49.5	41.3
Alcohol (g/week)	2.15 (1.03)	2.04 (1.02)	2.25 (1.03)
eGFR (ml/min/BSA)	89.1 (11.6)	88.3 (12.0)	90.0 (11.1)
Exercise habits (%)	Never: 28.0	26.4	29.8
	Occasionally: 21.9	23.0	20.7
	Once a week: 20.3	22.3	18.2
	2–3 times a week: 17.3	17.3	17.3
	> 3 times a week: 12.5	11.0	14.0
Non HDL-cholesterol (mmol/l)	3.8 (1.0)	3.8 (1.0)	3.9 (1.0)
HDL-cholesterol (mmol/l)	1.6 (0.5)	1.8(0.4)	1.4 (0.4)
BMI (kg/m^2^)	26.8 (4.3)	26.4 (4.7)	27.3 (3.8)
Systolic blood pressure (mmHg)	125.8 (16.8)	122.9 (17.6)	128.7 (15.5)
Diabetes (%)	7.2	4.9	9.6
Statin use (%)	6.6	5.3	7.9

Means and SD or proportions are given.

*BMI* body mass index, *HDL* High density lipoprotein, *eGFR* estimated glomerular filtration rate.

### Distributions of TropI and NT-proBNP

**Fig. 1:**
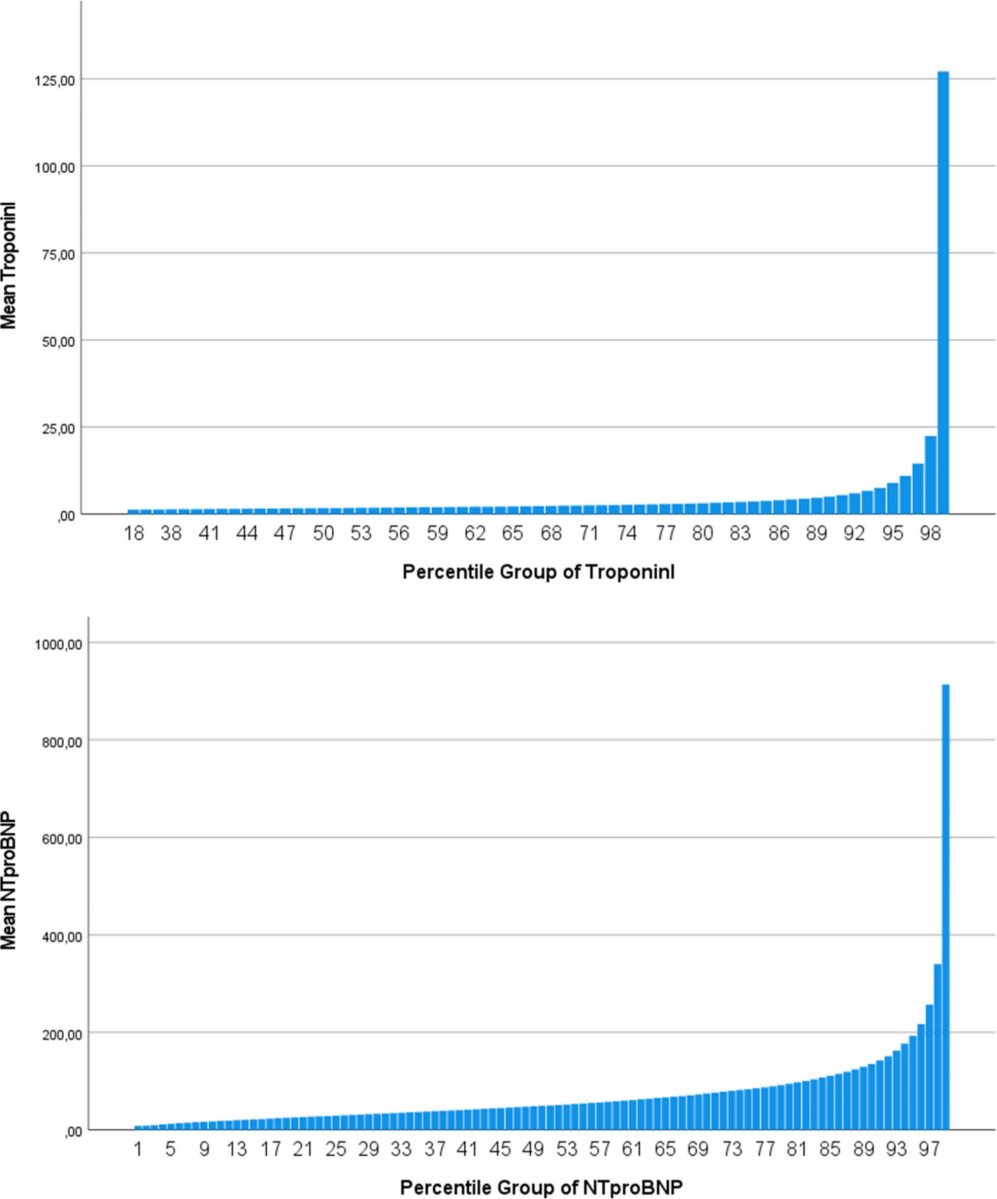
Mean values for troponin I and NT-proBNP in each percentile of the distribution.

The distribution of TropI and NT-proBNP levels in the sample is shown as mean values in each percentile of the distributions in Fig. 1. As can be seen, the levels were heavily skewed to the right for both biomarkers. Histograms are given in suppl Fig. 1.

37% of the sample showed TropI levels below LOD (1.3 ng/L). Individuals with TropI levels below LOD were defined as group one. The remaining distribution was divided into quartiles (groups two to five). The min and max in each group are given in suppl Table 1. Two subjects showed TropI levels almost 10 times higher (> 5000) than the third highest level. These two subjects were excluded from the analysis.

Only < 1% of the sample showed levels of NT-proBNP below LOD. This distribution was therefore divided into quintiles. The min and max in each group are given in suppl Table 1.

### SIS vs. TropI and NT-proBNP

**Fig. 2:**
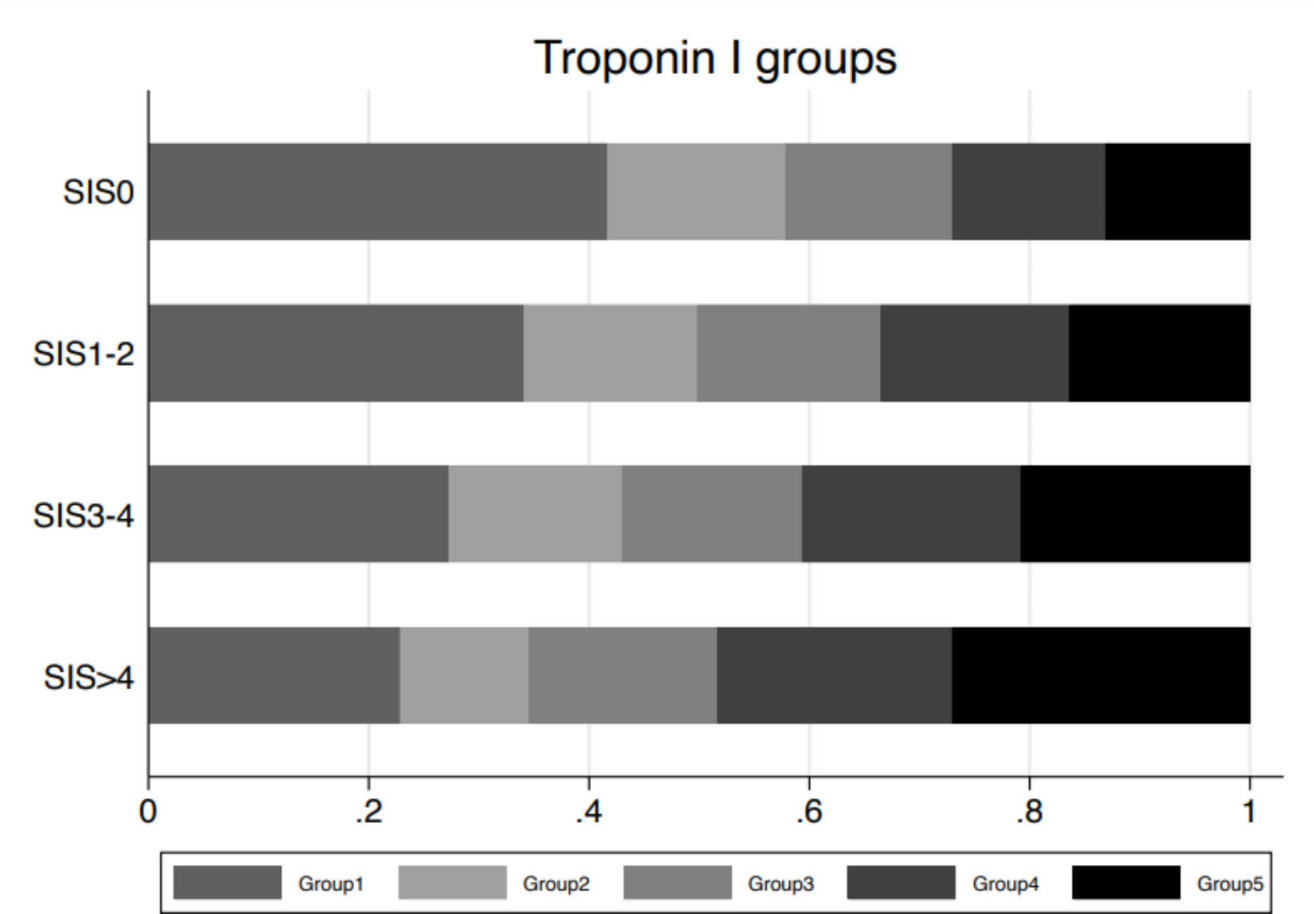
Proportion of troponin I level groups in relation to SIS groups. High levels of troponin I groups indicate high levels of troponin I, while high levels of SIS groups indicates increased amount of coronary atherosclerosis.

The median level of TropI increased with increasing SIS, from 1.5 ng/L in the SIS group without any atherosclerosis to 2.3 ng/L in the highest SIS group (see Suppl Table 2). Also, the proportion of high TropI groups (groups 4 and 5) increased with increasing SIS group (*p* < 0.0001, Fig. 2).

SIS was related to TropI both in the age, sex and site-adjusted model, as well as in the multiple adjusted model (Table 2). The strength of this association was similar in men and women (*p* = 0.87 for sex-interaction). As can be seen by the Wald z-value (as a measure of the importance in the model) in suppl Table 3, sex was by far the most important variable for TropI levels followed by systolic blood pressure and eGFR, while SIS showed an importance of the same magnitude as age, non-HDL-cholesterol and BMI. Other factors, such as diabetes, HDL-cholesterol, exercise habits and education levels, showed a lower z-value.

**Table 2:** Relationships between the segment involvement score (SIS) group (4 levels) and troponin I (TropI) group (5 levels) for two levels of adjustment in the full sample, and adjusted for age, and site in sex-stratified models.

Biomarker	Adjustment	OR	SE	Wald	*p*-value	95%CI
TropI (all subjects)	Age, sex, site	1.15	0.014	11.0	<0.001	1.12–1.18
TropI (all subjects)	Multiple	1.12	0.016	8.47	<0.001	1.09–1.16
TropI (men only)	Age, site	1.15	0.018	9.39	<0.001	1.12–1.19
TropI (women only)	Age, site	1.14	0.025	6.1	<0.0001	1.09–1.19
NT-proBNP (all subjects)	Age, sex, site	1.06	0.013	4.7	<0.001	1.03–1.08
NT-proBNP (all subjects)	Multiple	1.10	0.015	7.1	<0.001	1.07–1.13
NT-proBNP (men only)	Age, site	1.06	0.016	4.1	<0.0001	1.03–1.10
NT-proBNP (women only)	Age, site	1.03	0.022	1.5	0.120	0.99–1.07

OR represents the change in Odds ratio per one level increment in SIS category.

The multiple adjusted model included age, sex, study site, statin use, exercise habits, education level, alcohol intake, smoking status, diabetes, BMI, HDL-cholesterol, non-HDL-cholesterol, systolic blood pressure, and eGFR as confounders.

Adding NT-proBNP to this model showed that NT-proBNP was related to TropI levels (*p* < 0.001), but the estimate for SIS was only changed to a minor degree (now OR 1.12, 95%CI 1.08–1.15, *p* < 0.0001), suggesting that a failing heart is not driving the association between SIS and TropI.

When using TropI above the 99th percentile as a binary trait in logistic sex-stratified regression analyses, SIS was related to this TropI-trait in an age and site-adjusted model in men (OR 1.21, 95%CI 1.03–1.42, *p* = 0.019), but not in women despite a similar point estimate (OR 1.20, 95%CI 0.97–1.428 *p* = 0.087). 92 of the women and 42 of the men with no detectable coronary atherosclerosis showed levels above the sex-specific 99% for TropI.

In the subjects with TropI < LOD (37% of the sample), 34% showed SIS > 0 and 33% showed CACS > 0. Given that Trop > LOD is a positive test and SIS > 0 is the disease, the sensitivity for positive test to detect disease was 0.58. The specificity was 0.30. The positive predictive value (PPV) was 0.54 and the negative predictive value (NPV) was 0.34.

Contrary to TropI, the median level of NT-proBNP did not increase with increasing SIS, see suppl Table 2. Neither did the proportions of the highest NT-proBNP groups increase with increasing SIS group (suppl Fig. 2).

However, both when adjusted for age, sex and site and in the multiple-adjusted model, SIS was related to NT-proBNP, as can be seen in Table 2. The main correlates of high NT-proBNP in this study were sex and age, followed by blood pressure and non-HDL-cholesterol, as could be seen in suppl Table 4. SIS was related to NT-proBNP of the same order as diabetes, HDL-cholesterol, BMI, and eGFR.

A sex-interaction was seen for NT-proBNP (*p* = 0.012), and when the sample was divided by sex, a significant correlation between SIS and NT-proBNP was seen only in men.

When this relationship in males was adjusted for TropI levels, the SIS vs. NT-proBNP relationship was markedly attenuated and was now only borderline significant (OR 1.03, 95%CI 1.006–1.07).

### CACS vs. TropI and NT-proBNP

A similar picture emerged when CACS was related to the two cardiac biomarkers. CACS was related to TropI in both age, sex and site-adjusted models, as well as in multiple-adjusted models and this relationship was similar in men and women (*p* = 0.84 for sex-interaction).

NT-proBNP was also related to CACS, but as with SIS a significant sex-interaction was seen with less strong relations vs. CACS in women than in men (see suppl Table 5 for details).

### Degree of stenosis at each segment vs. TropI and NT-proBNP

**Fig. 3:**
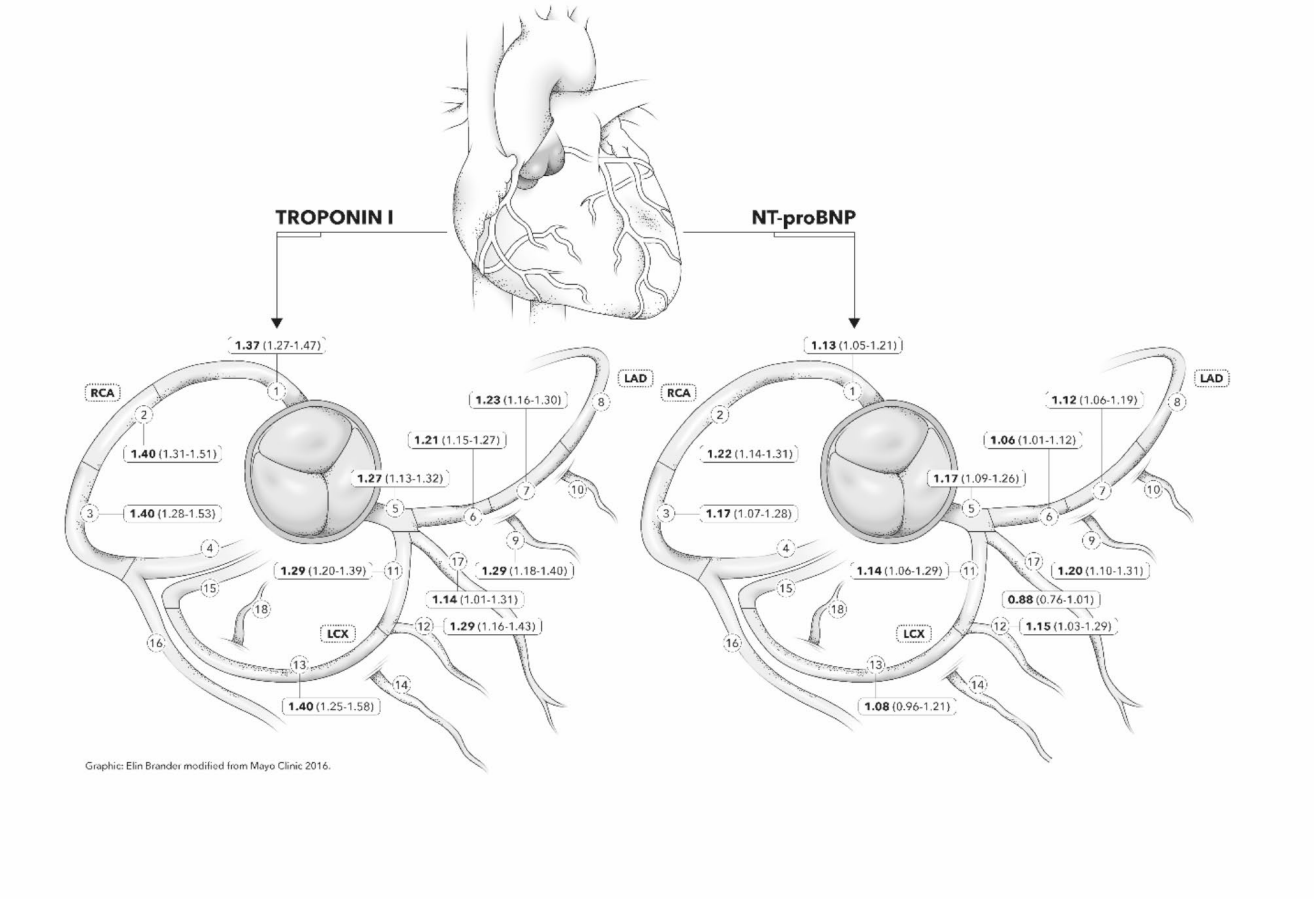
Relationships between degree of stenosis in 11 coronary artery segments vs. Troponin I (left side) or NT-proBNP (right side) levels given as odds ratios (OR, in bold) and 95%CI (in parenthesis). The numbering of the segments is according to^24^. Figure modified from Ayoub et al.^25^ used with permission of Mayo Foundation for Medical Education and Research, all rights reserved. The OR given in the figure are based on ordinal logistic regression for 0, 1–49% and > 50% stenosis in each of the coronary segments and are reported per category increment of the different biomarkers.

When the degree of stenosis in each coronary artery segment was categorized in three groups (no stenosis, 1–49% stenosis, ≥ 50% stenosis) and each of the 11 proximal segments were related to TropI levels, the degree of stenosis was strongly related to TropI in all evaluated segments after adjustment for age, sex and site (Fig. 3).). When proximal segments representative for all three coronary arteries (segments 1, 6 and 11) were entered into the same model, the degree of stenosis in all three segments were significantly related to TropI levels (*p* < 0.005 for all three). The highest estimate in this multiple-adjusted model was seen for segment 1 (right coronary artery, see suppl Table 6 for details).

No significant sex-interactions were seen for any of the 11 segments regarding TropI levels.

A similar pattern was seen for NT-proBNP, but in this case the estimates were generally lower than for TropI. When representatives for proximal segments for all three coronary arteries (segments 1, 6 and 11) entered the same model together with TropI levels, none of the segments were significantly related to NT-proBNP (all *p* > 0.05).

Significant sex-interactions regarding NT-proBNP were seen for segments 1, 2 and 7 with stronger relationships in men compared to women.

### Discrimination of coronary stenosis by use of TropI or NT-proBNP

TropI (or NT-proBNP) alone showed poor discriminatory power (ROC-AUC 0.5590 and 0.5343, respectively) regarding any coronary atherosclerosis. A very small, but significant, improvement in ROC-AUC was seen when these two biomarkers were added to age and sex (+ 0.0005 and + 0.0002, respectively).

These two biomarkers also improved the discriminatory power when added on top of the traditional risk factors used in the models above. Although significant (*p* = 0.019), these additions were very modest in terms of improvement in ROC-AUC (+ 0.0005 and + 0.0002, respectively). For details, see Table 3.

**Table 3:** Discrimination of coronary stenosis by use of troponin I (TropI) or NT-proBNP in comparison with age, sex and site, or the traditional risk factors: age, sex, study site, statin use, exercise habits, education level, alcohol intake, smoking status, diabetes, BMI, HDL-cholesterol, non-HDL-cholesterol, systolic blood pressure, and eGFR.

Variables in model	ROC-AUC	95% CI		Comparisons
Age and sex	0.7029	0.69637	0.70948	
TropI only	0.5590	0.55175	0.56627	
NT-proBNP only	0.5343	0.52696	0.54158	
Age and sex + TropI	0.7034	0.69687	0.70997	*P* = 0.0090 vs. age and sex
Age and sex + NT-proBNP	0.7031	0.69651	0.70962	*P* = 0.032 vs. age and sex
All risk factors	0.7386	0.73192	0.74528	
All risk factors + TropI	0.7391	0.73239	0.74575	*P* = 0.014 vs. all risk factors
All risk factors + NT-proBNP	0.7388	0.73215	0.74552	*P* = 0.018 vs. all risk factors
All risk factors + TropI + NT-proBNP	0.7392	0.73248	0.74584	*P* = 0.0019 vs. all risk factors

TropI did not significantly add any discriminatory power when added on top of the traditional risk factors when the dependent variable was any stenosis > = 50%.

Neither did TropI add any discriminatory power when added on top of SCORE, as a comprehensive marker of risk (ROC-AUC for SCORE only: 0.7090, for SCORE plus TropI: 0.7092, p-value 0.43).

## Discussion

The present study showed that the burden of coronary atherosclerosis at CCTA, as well as CACS, was associated to TropI levels in subjects without a history of atherosclerotic disease confirming our hypothesis. We also found that that lesions in the proximal segments of all three major coronary arteries contributed independently of each other. TropI levels did not improve discrimination of the presence of coronary atherosclerosis compared to traditional risk factors in any clinically important way. NT-proBNP levels were also related to burden of coronary atherosclerosis, especially in men, but with lower estimates than seen for TropI.

A link between the severity of coronary atherosclerosis and TropI levels has previously been described in cohorts that included CHD or chest pain patients, using either invasive angiography, CT angiography or CT-derived coronary calcium^7–11^. We could only find one study conducted in the general population using CACS with findings similar to the present study^12^. The novelty of our evaluation is that we used data from a large sample free from previously diagnosed atherosclerotic disease in which CCTA was performed along with CACS determinations. Taken together, it seems as if the relationship between the severity of coronary atherosclerosis and TropI levels is present both in groups with a high degree of coronary stenosis, such as in CHD patients, and in populations with a low degree of coronary atherosclerosis, such as in population-based studies of individuals without a history of atherosclerotic disease.

We used SIS as the primary exposure in the present study. SIS is a measure of the extent of atherosclerosis in the coronary circulation, but does not include information on the severity of stenosis. We therefore did an additional analysis for each segment using a grading of the degree of stenosis in three levels (no stenosis, 1–49% stenosis, ≥ 50% stenosis), an analysis that showed also the importance of the impact of the degree of stenosis in terms of TropI levels.

Another unique feature of the present study is the evaluation of coronary stenosis at the segment level. Since stenosis in the proximal segment 6 in LAD is most commonly seen in this sample^16^ and obstruction of LAD by a thrombus could result in profound TropI elevations, it could be thought that LAD would be the coronary artery contributing the most to the observed relations between the amount of coronary stenoses and TropI levels. However, as could be seen in Fig. 3, the ORs for different proximal segments vs. TropI levels were not highest for the LAD segments, and when representatives for proximal segments from all three major coronary arteries were used as exposures in the same multivariable model all of them were significantly related to TropI levels independently of each other. In fact, both when the segments were evaluated one by one, as well as in the multivariable model, the right coronary artery (segment 1) showed higher estimates than the LAD segments. Thus, the combined effect of stenosis in several segments, as evaluated by SIS, rather than stenosis in some specific segments, seems to be linked to TropI levels.

The mechanism whereby coronary stenosis would result in increased TropI levels in individuals without a history of CHD and no chest pain at the time of the investigation is not obvious. One explanation could be silent myocardial infarctions. In the PIVUS study, myocardial scars, indicative of a previous myocardial infarction, were detected in 20% of the sample free from a history of CHD using Gadolinium late-enhancement MRI^17^. Elevated TropI levels were found in this group^18^. However, this potential mechanism does not explain why 92 of the women and 42 of the men with no detectable coronary atherosclerosis showed levels above the sex-specific 99% for TropI in the SCAPIS sample. We found that a number of other factors, such as BMI, eGFR, systolic blood pressure and plasma lipids were associated with TropI levels^1,2^. Some of these factors are also related to atherosclerosis and could thereby be common causes of coronary atherosclerosis and raised TropI levels. Indeed, SBP was a major determinant of raised TropI level, and both eGFR and BMI were related to TropI levels with the same magnitude as SIS in the multiple-adjusted model (suppl Table 3). The impact on the heart of such risk factors has been suggested to be a part of the concept of chronic myocardial injury, as reviewed by Chapman et al.^19^. Another mechanism not evaluated in the present study is coronary microvascular dysfunction, which might lead to elevated TropI levels^20^.

Contrary to findings in the Danish population-based study^12^, we could not demonstrate that the addition of TropI to the traditional cardiovascular risk factors improved discrimination of any atherosclerosis to any clinically meaningful degree, although the minor increase in C-statistics was statistically significant. One difference between the studies is the use of CACS in the Danish study and the addition of CT angiography in the SCAPIS study.

It is known from epidemiological studies that also apparently healthy individuals could have elevated TropI levels over time (1). The present study shows that the risk of coronary stenosis is elevated with elevated TropI levels also in subjects without a history of atherosclerotic disease. If it is worthwhile to perform a coronary CT scan in the individuals with elevated TropI levels over time would be an important question to evaluate in future studies.

We also evaluated NT-proBNP in the same fashion as TropI and found a weaker relationship with coronary atherosclerosis than for TropI. Furthermore, an interaction with sex was found with a stronger relationship in men than in women, with no significant relationship in the latter. Since it is plausible that myocardial ischemia could be a mediator between coronary atherosclerosis and elevated NT-proBNP levels, we further adjusted for TropI as a marker of ischemia, and found that the relationship between SIS and NT-proBNP was markedly attenuated. Although this was not a formal test of mediation, this supports the idea that myocardial ischemia could be an important mediator between coronary atherosclerosis and elevated NT-proBNP levels.

The major strength of the present study is the large sample size with CT coronary angiography data derived from the general population. One limitation is that the vast majority of subjects included were of European descent, and therefore the results from this study have to be confirmed in other ethnic groups in order to be judged to be general. Another limitation is that about a third of the population showed TropI levels below LOD and therefore forced us to use a grouped variable for TropI, rather than using TropI as a continuous variable. If anything, however, using TropI as a categorical variable should likely result in a somewhat reduced statistical power. In this study we find an association between TropI levels and the burden and severity of coronary atherosclerosis. However, since the progression of coronary atherosclerosis to an overt myocardial infarction is dependent on many other factors, such as the vulnerability of a plaque and hemostatic factors, it is not automatically given from our findings evident that TropI levels are related to an increased risk of myocardial infarction.

In conclusion, the burden of coronary artery atherosclerosis was related TropI levels in both men and women, and all three major coronary arteries contributed to this relationship. However, adding TropI to traditional risk factors did only marginally increase discrimination of atherosclerosis.

## Methods

### Study sample

The SCAPIS study enrolled 30,154 individuals aged 50–65 years (51% women) in six cities in Sweden during 2013–2018 (predetermined sample size 30,000). The invitation was given in a random fashion by analogue mail. The participation rate was 50%. The details on recruitment have been described in a previous publication^15^. The present study sample consisted of 25,859 subjects with valid examinations of CCTA together with valid TropI determinations following exclusion of 1,066 subjects with either a self-reported history of myocardial infarction, stroke, angina pectoris, coronary revascularization, or other diagnosed atherosclerotic stenosis, such as peripheral arterial disease.

The present study used a cross-sectional design.

The study was performed according to relevant guidelines and regulations (declaration of Helsinki and following updates). The SCAPIS study was approved by the Ethics Committee of Umeå University; Sweden (Nr 2010-228-31 M). All participants provided written informed consent.

### Investigations

#### Risk factors

Blood samples were drawn after an over-night fast. Glucose, total cholesterol, HDL-cholesterol and triglycerides were measured by standard techniques at the different sites. Non-HDL-cholesterol was defined as total cholesterol minus HDL-cholesterol. Blood pressure was measured twice in both arms after 5 min of rest in the supine position, using an Omron M10-IT automated oscillometric device. The mean value of the two measurements for the arm with the highest mean systolic blood pressure was used. Height and weight were measured by standard techniques, and body mass index (BMI) was calculated (weight/height^2). Glomerular filtration rate (eGFR) was calculated from determinations of serum creatinine using the modified CKD-EPI formula^21^. Diabetes was defined as a previous diagnosis of diabetes or a fasting plasma glucose measurement of > = 7.0 mmol/l at the examination.

#### Lifestyle factors

Information on lifestyle, medication and medical history was obtained by a questionnaire. Smoking was defined on a three-level scale (never, previous or current smoker). Alcohol intake was determined in grams/week. Exercise habits were given on a scale with five levels, 0 = Never exercise, 1 = Only occasionally exercise, 2 = 1–2 times a week, 3 = 2–3 time a week, 4 = More than 3 times a week. Education was defined on a three-level scale (< 10, 10–12, > 12 years in school).

#### CCTA

Cardiac imaging in SCAPIS has been described in detail previously^16^. Briefly, CT was performed using a dedicated dual-source CT scanner equipped with a Stellar Detector (Somatom Definition Flash, Siemens Medical Solution, Forchheim, Germany). In preparation for CCTA imaging, renal function was assessed and potential contraindications identified to exclude participants for whom administration of contrast media could pose a risk. A β-blocker (metoprolol) and sublingual glyceryl nitrate were given for control of heart rate and dilation of coronary arteries. The contrast medium iohexol (GE Healthcare, 350 mg I/mL) was administered at a dose of 325 mg I/kg body weight. CCTA was performed at 100 or 120 kV using five different protocols depending on heart rate, heart rate variability, presence of calcifications, and body weight.

After reconstruction of images, all 18 coronary artery segments were visually examined for the presence of plaques as described^16^. Per-segment status of the coronary vessel was defined as: no atherosclerosis; 1–49% stenosis; ≥50% (i.e., significant) stenosis. Luminal obstruction was defined by visually estimating diameter stenosis (using the average of the longest and shortest diameter at the site of stenosis). Segments not assessable because of calcium blooming were coded as 1–49% stenosis and segments with technical failures were coded as missing data. Individuals were classified based on the highest degree of stenosis present in the coronary artery circulation. In addition to degree of stenosis, extent of atherosclerosis in the coronary tree was calculated for each individual as the sum of coronary segments with atherosclerosis (segment involvement score, SIS)^22^.

All non-contrast image sets were reconstructed and coronary calcium was identified and scored using the syngo.via calcium scoring software (Siemens, Erlangen, Germany). The area of calcification of each 3 mm slice was multiplied with an intensity factor and summed up to a coronary artery calcium score (CACS) for the artery tree according to Agatston^23^.

The numbering of the segments follows the 18 coronary segment model defined by the Society of Cardiovascular Computed Tomography^24^.

#### Measurements of TropI and NT-proBNP

TropI and NT-proBNP were analysed from frozen samples of fasting EDTA-plasma, using an Abbott Alinity I analyzer. Alinity I STAT hs Troponin I and Alere NT-proBNP reagents were used. Limit of detection (LOD) was 1.3 ng/L for TropI and 8.3 ng/L for NT-proBNP.

### Statistical analysis

For the initial assessment of the associations between the dependent variables TropI and NT-proBNP with respect to age and sex, values below LOD were treated as LOD. The non-parametric Spearman’s rank correlation and Mann-Whitney U-test were used.

To become comparable, the distributions of both TropI and NT-proBNP were divided into 5 groups. SIS was considered to be the primary exposure variable (being divided into 4 groups (0, 1–2, 3–4, > 4).

In the analysis of the primary aim, ordinal logistic regression analyses with groups of TropI as dependent variable and SIS as independent variable were performed. Two levels of adjustment were performed. First, we adjusted for age, sex and study site. Second, in the multi-adjusted model further adjustment was made for statin use, self-reported exercise habits, education level, alcohol intake, smoking status, diabetes, BMI, HDL-cholesterol, non-HDL-cholesterol, systolic blood pressure and eGFR were added as potential confounders. Since missing data on confounders were uncommon, listwise deletion was used.

To investigate if the associations differed between men and women, an interaction term between SIS and sex were added in a separate model.

Similar analyses were also carried out using CACS (being divided into 4 groups (0, 1–99, 100–299, ≥ 300) as a secondary exposure variable.

We also investigated how the degree of stenosis in different coronary artery segments might influence TropI levels in an exploratory analysis. Each coronary artery segment was divided in three groups (no stenosis, 1–49% stenosis, ≥ 50% stenosis). Ordinal logistic regression analysis with groups of TropI as dependent variable and the degree of stenosis as independent variable (together with age, sex and site) was performed for 11 proximal coronary segments.

To investigate if TropI levels could be used to improve the prediction of coronary atherosclerosis (binary; absent or present), we used logistic regression analysis with any atherosclerosis as dependent variable. The main analysis was to evaluate if addition of TropI to the risk factors used in the multi-adjusted model described above would increase the discrimination of any atherosclerosis compared with the risk factors used in the multi-adjusted model. For this purpose, we used C-statistics to compare the models.

All above mentioned analyses were also carried out using NT-proBNP as the outcome variable.

STATA 16.1 was used for the calculations.

## Electronic supplementary material

Below is the link to the electronic supplementary material.

Supplementary Material 1

## Data Availability

The data underlying this manuscript cannot be shared publicly for legal regulations related to the privacy of individuals that participated in the study. The data will be shared on reasonable request to the corresponding author.
