# Supplementary material for: Cardiac biomarkers for detection of coronary artery disease in the community
Source: Sci Rep. 2024 Dec 16;14:30514. doi: 10.1038/s41598-024-82777-x (PMC11649811; doi:10.1038/s41598-024-82777-x)
Supplement: Supplementary file 1 — Supplementary Material 1 [file 41598_2024_82777_MOESM1_ESM.docx]

**Suppl table 1.** **Division of troponin I and NT-proBNP in five groups.** Lowest and highest values in each group are given.

-------------------------------------------------------

Troponin I|

| N min max

----------+--------------------------------------------

1 | 10,452 <1.3 <1.3

2 | 4,454 1.31 1.76

3 | 4,445 1.77 2.31

4 | 4,442 2.32 3.57

5 | 4,443 3.58 651.9

NT-proBNP |

| N min max

----------+--------------------------------------------

1 | 5,640 8.3 25.43

2 | 5,640 25.44 39.77

3 | 5,638 39.81 57.74

4 | 5,634 57.76 89.53

5 | 5,638 89.56 5062.42

-------------------------------------------------------

**Suppl table 2.** Levels of troponin I and NT-proBNP in different SIS groups. IQR=interquartile range.

Troponin I

--------------------------------------------------------------------------------

SIS group | min low IQR median high IQR max

----------+---------------------------------------------------------------------

0 | 1.3 1.3 1.53 2.41 651.9

1 | 1.3 1.3 1.77 2.76 465.0

2 | 1.3 1.3 2 3.2 589.7

3 | 1.3 1.41 2.28 3.85 338.7

Nt-proBNP

--------------------------------------------------------------------------------

SIS group | min low IQR median high IQR max

----------+---------------------------------------------------------------------

0 | 8.3 29.49 48.42 79 3876.84

1 | 8.3 27.22 44.84 75.43 2758.83

2 | 8.3 27.82 45.67 78.64 5062.42

3 | 8.3 27.49 46.17 81.65 4029.07

**Suppl table 3.** Relationship between the SIS group (4 levels) and troponin I groups (5 levels) in the fully adjusted model. A p-value of 0.000 denotes <0.001.

| Odds Ratio Std. Err. z P-value 95% Conf. Interval

------------------+----------------------------------------------------------------

SIS | 1.129168 .0161311 8.50 0.000 1.09799 1.161232

Age | 1.024859 .003088 8.15 0.000 1.018824 1.030929

Female sex | .3769407 .0110552 -33.27 0.000 .3558839 .39924

Smoking |

former | .873553 .0229877 -5.14 0.000 .8296402 .9197902

current | .7403237 .0384106 -5.80 0.000 .6687416 .819568

Education | 1.094455 .0219275 4.50 0.000 1.052311 1.138287

Alcohol | .9863094 .0123989 -1.10 0.273 .962305 1.010913

eGFR | .3394735 .0318426 -11.52 0.000 .2824641 .4079891

Exercise | 1.007906 .0016753 4.74 0.000 1.004628 1.011195

NonHDLcholseterol | 1.106562 .0140388 7.98 0.000 1.079386 1.134423

HDL | 1.166096 .0374823 4.78 0.000 1.094899 1.241924

BMI | 1.023981 .0033037 7.35 0.000 1.017526 1.030476

Diabetes | 1.093796 .0562406 1.74 0.081 .9889389 1.209772

Statin use | .9087273 .0492165 -1.77 0.077 .8172081 1.010496

SBP | 1.015242 .000818 18.78 0.000 1.01364 1.016847

------------------+----------------------------------------------------------------

BMI=body mass index, HDL= High density lipoprotein, eGFR=estimated glomerular filtration rate, SBP=systolic blood pressure.

**Suppl table 4.** Relationship between the SIS group (4 levels) and NT-proBNP groups (5 levels) in the fully adjusted model. SBP= systolic blood pressure. GFR=estimated glomerular filtration rate. A p-value of 0.000 denotes <0.001.

----------------------------------------------------------------------------------

| Odds Ratio Std. Err. z P>|z| [95% Conf. Interval]

------------------+----------------------------------------------------------------

SIS | 1.0809 .0155181 5.42 0.000 1.050909 1.111747

Age | 1.072698 .0032103 23.45 0.000 1.066424 1.079008

Female sex | 3.30296 .0964398 40.92 0.000 3.119248 3.497491

Smoking |

previous | .9497684 .0246194 -1.99 0.047 .9027206 .9992682

currrent | 1.240418 .063001 4.24 0.000 1.122885 1.370253

Education | .8947641 .0177008 -5.62 0.000 .8607352 .9301383

Alcohol | 1.010396 .0125403 0.83 0.405 .9861143 1.035276

eGFR | .6508666 .0598465 -4.67 0.000 .5435318 .7793977

Exercise | 1.001741 .0015736 1.11 0.268 .998662 1.00483

Nonhdlcholseterol | .7998874 .010138 -17.62 0.000 .7802619 .8200064

HDL | 1.203308 .0378367 5.89 0.000 1.131388 1.279799

BMI | .9803063 .0031501 -6.19 0.000 .9741516 .9864999

Diabetes | .7402999 .0387964 -5.74 0.000 .6680353 .8203818

Statin use | .7059219 .0384502 -6.39 0.000 .634444 .7854526

SBP | 1.013922 .0008075 17.36 0.000 1.01234 1.015506

BMI=body mass index, HDL= High density lipoprotein, eGFR=estimated glomerular filtration rate, SBP=systolic blood pressure.

**Suppl table 5.** Relationships between the coronary artery calcium score (CAC) group (4 levels) and troponin I (TropI) group (5 levels) for two levels of adjustment in the full sample and adjusted for age, and site in sex-stratified models.

| Biomarker | Adjustment | OR SE. Wald p-value 95%CI |
| --- | --- | --- |
| TropI (all subjects) | Age, sex, site | 1.17 .016 11.2 <0.001 1.13-1.20 |
| TropI (all subjects) | multiple | 1.14 .018 8.6 <0.001 1.11-1.18 |
| TropI (men only) | Age, site | 1.18 .020 9.5 <0.001 1.14-1.22 |
| TropI (women only) | Age, site | 1.15 .027 6.1 <0.001 1.10-1.21 |
|  |  |  |
| NT-proBNP (all subjects) | Age, sex, site | 1.07 .015 5.2 <0.001 1.04-1.10 |
| NT-proBNP (all subjects) | multiple | 1.09 .017 5.6 <0.001 1.06-1.12 |
| NT-proBNP (men only) | Age, site | 1.08 .019 4.3 <0.001 1.04-1.11 |
| NT-proBNP (women only) | Age, site | 1.05 .025 2.0 0.038 1.002-1.10 |

**Suppl table 6.** Relationships between troponin I levels (5-group scale) and degree of stenosis (3-graded scale) in three coronary artery segments chosen to represent proximal segments in all three coronary arteries. Dx=right coronary artery. LAD=Left anterior descending artery. LCX: Left circumflex artery. The numbering of the segments is according to (20).

------------------------------------------------------------------------------

| Odds Ratio Std. Err. z P-value [95% Conf. Interval]

-------------+----------------------------------------------------------------

Dx:seg1 | 1.246729 .0503823 5.46 <0.001 1.151791 1.349492

LAD:seg6 | 1.111979 .0315206 3.74 <0.001 1.051884 1.175507

LCX:seg11 | 1.124666 .0466620 2.83 0.005 1.03683 1.219943

**Suppl figure 1.** **Distribution of Troponin I and NT-proBNP levels in the SCAPIS sample (using 100 bins).** Two subjects with Troponin I levels >5000 ng/L (being extreme outliers) were excluded from the analyses.


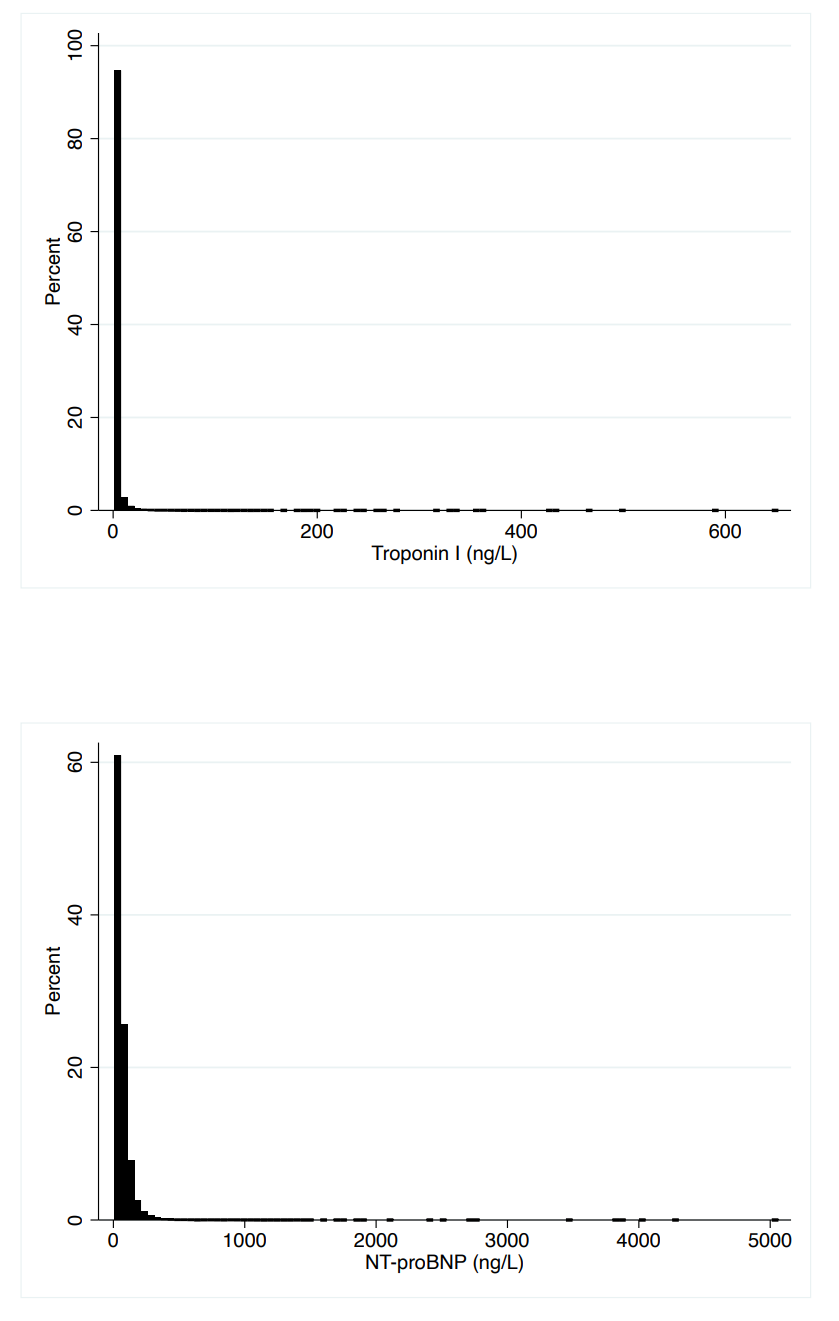


**Suppl figure 2.** Proportion of NT-proBNP level groups in relation to SIS groups. High levels of troponin I groups indicate high levels of troponin I, while high levels of SIS groups indicates increased amount of coronary atherosclerosis.
